# Antimicrobial resistance as a super wicked problem: how do we engage the public to be part of the solution

**DOI:** 10.1016/j.infpip.2023.100314

**Published:** 2023-11-14

**Authors:** Helen Rickard, Sam Watkin, Nicola Baldwin, Anthony De Souza, Lena Ciric, Elaine Cloutman-Green

**Affiliations:** a Healthy Infrastructure Research Group, University College London, Department of Civil Environmental and Geomatic Engineering, Chadwick Building, London, UK; b Nosocomial Project, London, UK; c Great Ormond Street Hospital NHS Foundation Trust, Camelia Botnar Laboratories, Department of Microbiology, London, UK

**Keywords:** Public engagement, Patient involvement, Antimicrobial resistance, Outreach, Behaviour change, Education

## Abstract

Antimicrobial resistance (AMR) is now regarded as one of the greatest global challenges of the 21^st^ century. The complexity, urgent timeframe, and lack of clear solution to AMR have contributed to its classification as a ‘super wicked problem’. Yet knowledge surveys of the general public have found that they still harbour numerous misconceptions linked to both the sources and impact of AMR. This confusion is compounded by AMR being a One Health issue, and therefore a factor in not just human health but in other industries, such as farming. This can further inhibit understanding and knowledge transfer around AMR for those without a prior knowledge base.

In order to address the escalating risk that AMR presents, however, it is essential to address this knowledge gap and engage with the public to support wide scale changes in behaviour and consumer choice. The WHO now requires national action plans tackling AMR to include patient and public involvement/engagement (PPI/E) to support changing the trajectory of AMR. Despite this, little detail is available as part of strategic plans on how PPI/E should be undertaken in order to aid implementation. This paper discusses a number of approaches to support the design and delivery of PPI/E in relation to AMR, including the different social behaviour models underlying successful PPI/E strategies, and key considerations linked to specific activity types. The framework produced includes features for steps from initial planning and design through to evaluation. The aim is to help improve the ability of scientists and healthcare professionals to produce high quality AMR PPI/E.

## Introduction

Antimicrobial resistance (AMR) was estimated to contribute to 1.27 million deaths in 2019 and is expected to result in 10 million deaths per year by 2050 [1]. Despite these implications for human health the behaviour change required to prevent escalation within both healthcare workers and in the general population is proving difficult to achieve. Global antibiotic consumption is increasing exponentially with poor public understanding of what AMR is and its potential impact [2], with one study reporting only 9% of participants understanding that AMR occurs in bacteria not the host [3].

One obstacle to addressing AMR as an increasingly serious issue, is that it is a ‘super wicked problem’. Super wicked problems typically combine inherent complexity, with numerous interrelated biological and social drivers. Multiple stakeholders impede a single agency taking charge, with limited time available to reduce potential impact [1]. AMR is also a One Health engagement challenge: with key stakeholders existing beyond the area of human health, including within the farming and veterinary sectors.

Within human health, usage of antimicrobials is influenced by the knowledge, attitudes, and beliefs of both healthcare workers and the public [2]. It is estimated that historic public AMR campaigns between 1997 and 2007 across Europe led to the equivalent of a 6.5–28.3% drop in the mean level of antibiotic use [4]. The WHO has therefore acknowledged the importance of including members of the public within strategic planning in order to change the trajectory of AMR, with a requirement to include public engagement within all national action plans (NAPs). Although many NAPs recognise that public education raises awareness of AMR, few include strategic pathways for implementation.

Undertaking patient and public involvement/engagement (PPI/E) is a recognised key pathway to improving education and awareness, but also in supporting behaviour change within the public and healthcare professionals. The National Institute of Health Research defines patient and public involvement (PPI) as research done with or by patients and the public, not to, about, or for them. Involvement aims to work collaboratively with patients and the public including them in shared decision-making. Meanwhile, patient and public engagement (PPE) focuses on raising awareness and sharing research knowledge and findings; to tackle AMR as a ‘super wicked problem’, both approaches are required.

## Published approaches to PPI/E

Published evaluated examples of PPI/E activities and interventions encompass material or meetings delivered in various ways. Common examples include: face to face training, brochures, advertisements and billboards, educational videos, social media messaging, media engagement via interviews or films/TV, and educational conferences or science festivals [5].

Several high-profile approaches to AMR engagement have been evaluated and published including the Antibiotic Guardian Campaign and European Antibiotic Awareness Day. The Antibiotic Guardian campaign launched in 2014 using a One Health approach to change behaviour through collective action via the use of pledges [6]. The first European Antibiotic Awareness Day was launched in 2008 and consisted of 32 European countries sharing content distributed in 25 languages, with the first World Antibiotic Awareness week launched in 2015. These have demonstrated a focus on social media dissemination of material designed by focus groups [7] with campaigns targeting large scale distribution of information; which may require individuals to have a pre-existing interest in order to trigger engagement with either webpages or social media strategies.

Science, technology, engineering, arts and mathematics (STEAM) approaches, such as comic books, gamification or theatre, have become increasingly common [8]. While these have different advantages, the use of STEAM can support some of the more difficult conversations linked to AMR, such as managing individual versus community-level need, as they engage with emotion-based drivers in a way that allows participants to interact with information without obstacles such as fear from the apocalyptic aspects, or blame linked to prior personal choices [9]. Emotion has previously not been widely considered when designing or evaluating interventions; its growing use acknowledges that people are not driven by facts alone, but also by patterns of emotional response and ideological factors or beliefs [9].

**Figure 1:**
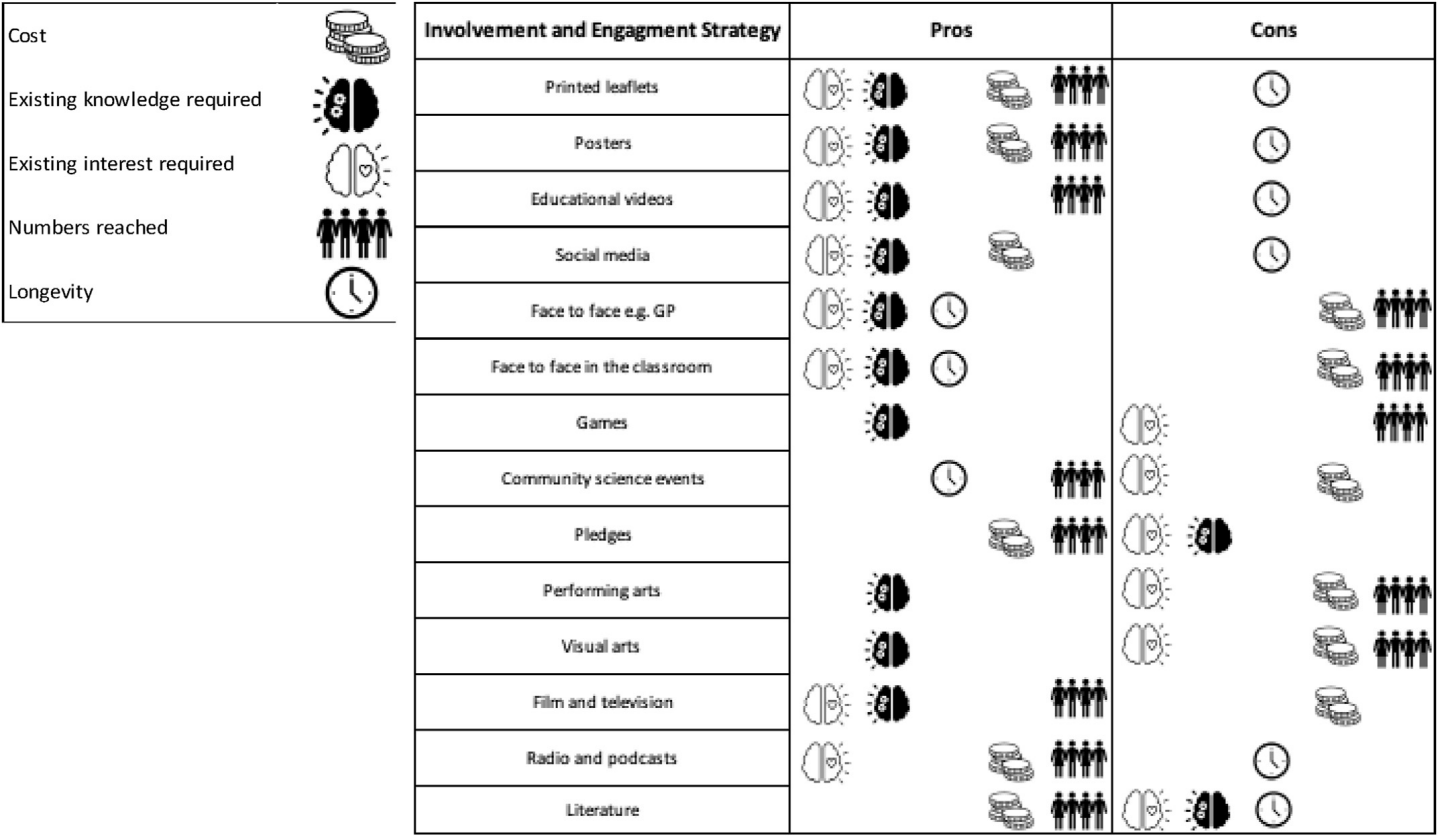
Advantages and disadvantages of AMR involvement and engagement activities. Figure 1 describes key factors to take into consideration during the selection of PPI/E approach. These factors include: resource availability (financial/time), reach/audience size anticipated, and prior knowledge of the problem required for engagement. As different activities are delivered across an audience spectrum they will combine these considerations in different ways. Examples of inclusion and engagement strategies and their respective advantages and disadvantages were derived from Redfern et al., 2020 [5].

Cognisant selection of the most appropriate approach for the outcome sought in conjunction with these factors should be undertaken (Figure 1). The PPI/E approach selected also drives the choice of evaluation tool to determine success and impact, and so evaluation methods should be derived from the activity selected.

## Social behaviour models

A review of published manuscripts demonstrates that few planned AMR PPI/E interventions were developed with behavioural theory embedded during the design, but were retrospectively mapped onto pre-existing theoretical constructs [10]. As most AMR interventions are not undertaken by social scientists, this approach can undermine meaningful evaluation or delivery of behavioural, rather than education-based impacts.

Common objectives amongst published interventions included shaping knowledge, impacting beliefs, changing environment, and improving effectiveness of professional roles on social influencing [10]. There are multiple theories available to support improved delivery of these interventions, with heterogeneous features to be considered when selecting the most appropriate approach, and thus support planning, delivery and evaluation.

Participatory learning and action approaches (PLA) are based around engaging community stakeholders in action-orientated, creative projects such as film, visual arts, photography, in order to produce meaningful change. Action orientated approaches are often introduced in synergy with education-based intervention to empower participants. This offers a bottom-up approach where communities are engaged to generate locally appropriate solutions. Linked to local engagement this aims to generate sustainable community wide changes in knowledge and behaviour by encompassing local social, cultural and economic factors [1].

Information-Motivation-Behavioural (IMB) skills approaches assume that behaviour in relation to health interventions is dependent on individuals being informed about the behaviour and its impacts, having the motivation to engage with changing behaviour, and adequate skills to effect change [11]. This approach can be valuable in determining what age groups and knowledge level are required for an intervention. It can help support innovation by acknowledging the differences that may be required to undertake PPI/E with, for instance, primary school children versus undergraduates. Motivational assessment can also consider emotional drivers in relation to behaviour change, and how these can be embedded within the intervention.

Transtheoretical approaches are based on the concept that cognition (awareness and knowledge) is required to achieve behaviour change. Therefore, interventions that disregard cognition will have limited sustainability and longevity, and therefore impaired long-term impacts. This theory encourages developing awareness of where the target participants are on the 6-stage intentional behaviour change spectrum (precontemplation, contemplation, preparation, action, maintenance and termination of previous behaviours) and designing interventions based on the surveyed knowledge, with an aim to move between phases and re-visit and modify interventions as appropriate [2].

## Planning your PPI/E

There are several different stages in planning the PPI/E activity, and decisions around planning interplay with whichever social behavioural model may be the most appropriate.

**Stage one:** Define the outcome/objective of the intervention and associated success criteria.

The main decisions during this stage are linked to whether the activity/intervention will be targeting education or behaviour or both. Even if the target is behaviour change alone, the approach may still need to undertake some aspects of knowledge increase, as behaviour change theories typically rely on different levels of pre-existing knowledge for the intervention to succeed [2]. Education based approaches may also fail to deliver anything but transient change if they disregard the need for contextualisation linked to individuals' own circumstances, communities, and pre-existing beliefs.

**Stage two:** Define the target audience and identify factors which may impact intervention efficacy.

Factors to consider that may define your audience and influence impact include [12]:

- Gender distribution
- Geographical location
- Age
- Family status (care givers, children)
- Professional status
- Socio-economic status
- Education level
- Shared experiences (special interest groups, prior intervention experience)
- Social and cultural norms (beliefs or religious practices)

**Stage three:** Select the most appropriate PPI/E activity.

**Figure 2:**
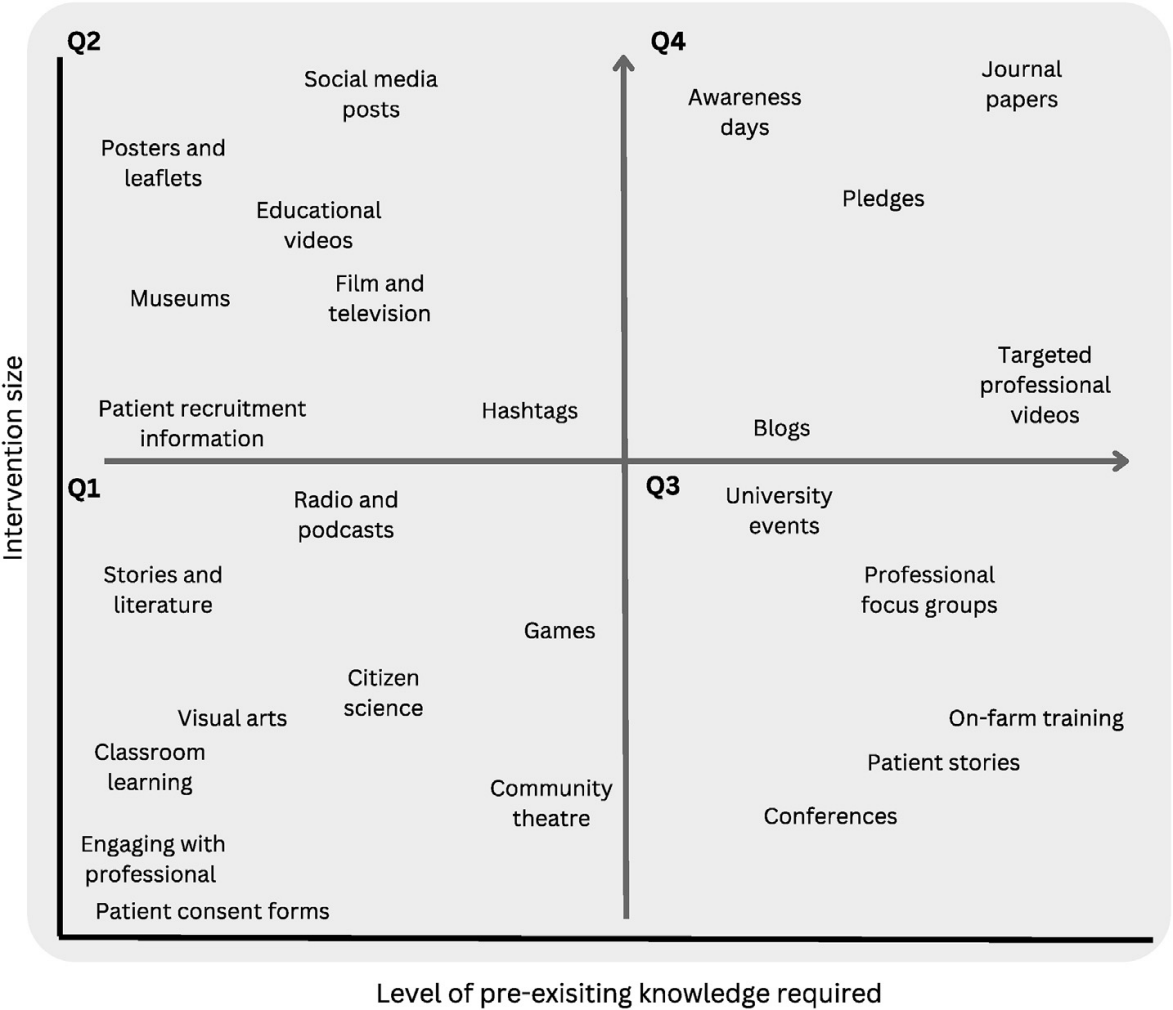
PPI/E activities in relation to numerical audience reach and prerequisite knowledge required. Data points on the graph represent specific engagement and inclusion activities, each point's position is determined by the combination of the knowledge level required for the activity and the size of the intervention. Quadrant 1 – low knowledge, small intervention, quadrant 2 – low knowledge, large intervention quadrant 3 – high knowledge, small intervention, quadrant 4 – high knowledge, large intervention. Quadrant location also takes into account whether any interaction will be self-directed, i.e. individuals seeking out the resource, or curated, where individuals are actively recruited or sought.

The combination of intervention outcome and selected target audience will guide what type of activity might be the most appropriate (Figure 2). If the target outcome is behavioural change, it is often difficult to achieve with a single stage intervention; multistage interventions however have resource implications [12]. As shown in Figure 2, key factors for activity selection requires acknowledgement of both the potential longevity of the impact targeted and the numerical reach of the activity. Activities such as those listed in quadrant one are more appropriate for smaller audiences with limited prior knowledge and understanding, in comparison those listed in quadrant four are more suitable for larger audiences who already have an understanding of the topic. These factors are impacted by not only the activity selected but the mechanism of delivery and the pre-existing knowledge or engagement within the target audience (see Figure 1).

It is often challenging to get high levels of engagement, leading to longevity of impact, with an approach that will reach a large number of individuals. High impact activities require a level of personalisation that can be challenging to attain via social media or large-scale communications. The right choice of intervention will also require consideration of sustainability, resource availability, acceptability and scalability, with different options likely to be appropriate at different stages of engagement [12].

**Stage four:** Design intervention evaluation tools.

Evaluation tools should be designed with measurement of the identified outcome in mind [12]. These tools are particularly important if the intervention is multi-stage or if it will be repeated, even if the activity is single stage however, evaluation should still be undertaken. For education style interventions, evaluation tools are frequently more straight forward, as they can be based on before-and-after knowledge assessments. Evaluating behavioural interventions may be more challenging as beliefs can be difficult to capture without bias, and long term follow up is required to establish the longevity of the intervention.

## Barriers to consider

Messaging and communication around AMR are considered to be a ‘Goldilocks’ problem, where messaging is required to be ‘just right’ in order to be successful. Mis-managed messaging can actually increase confusion and widen knowledge gaps, leading to a possible increase in health inequalities in certain communities. If incorrect, communication can then lead to fear, avoidance and reduced engagement. Individuals may feel blamed by the media or politicians and therefore reciprocal blaming of experts and systems can develop [13]. Another danger with AMR communication is that it can appear to address everyone, and therefore no one in particular, thus losing its impact [14].

Additionally health communication messaging is not linear; experts may construct and transmit the message, but it will be interpreted by the recipients in the light of pre-existing experience, knowledge and beliefs, embedded evaluation and feedback are therefore key [13]. Health systems can also impact reception of AMR messaging, both by healthcare professionals fearing negative reviews and members of the public participating as part of a consumer-based healthcare system. Designing activities within these settings may need to consider consumer attitudes, as well as traditional audience factors [15].

Finally, AMR interventions occur within an environment of ethical complexity, with intragenerational and intergenerational challenges. The interrelation of individual versus collective responsibility may be even more complex; for instance interventions linked to AMR have encountered challenges in implementation of behaviour change when parents engaged with healthcare interactions linked to their child's wellbeing, rather than their own [2]. Inequalities increase the risk that some people may suffer more from limited access to antimicrobials than others, especially those in low to middle income countries through either health access or farming. The ethics of antimicrobial access are clearly complex, with and without AMR considerations, and these need to be considered when designing any PPI/E activity [14].

## Conclusions

PPI/E is increasingly acknowledged as key to addressing AMR as a super wicked problem and has proven to be an effective method of gathering both behavioural change and educational awareness. Despite this, design of activities and interventions can be demanding with multiple factors to consider. As experts in the subject of AMR, we do not always possess the requisite skills in pedagogy or behaviour change to deliver interventions with maximum impact. We therefore need to learn from or collaborate beyond our disciplinary silos to implement the vital change we aspire to achieve.

## Conflict of interest statement

None declared.
